# The Impact of Head Model Choice on the In Vitro Evaluation of Aerosol Drug Delivery

**DOI:** 10.3390/pharmaceutics14010024

**Published:** 2021-12-23

**Authors:** Lauren Gallagher, Mary Joyce, Barry Murphy, Marc Mac Giolla Eain, Ronan MacLoughlin

**Affiliations:** 1 Research and Development, Science and Emerging Technologies, Aerogen Limited, Galway Business Park, H91 HE94 Galway, Ireland; lgallagher@aerogen.com (L.G.); mjoyce@aerogen.com (M.J.); bmurphy@aerogen.com (B.M.); mmacgiollaeain@aerogen.com (M.M.G.E.); 2 School of Pharmacy & Biomolecular Sciences, Royal College of Surgeons in Ireland, D02 YN77 Dublin, Ireland; 3 School of Pharmacy and Pharmaceutical Sciences, Trinity College, D02 PN40 Dublin, Ireland

**Keywords:** head model, aerosol drug delivery, facemask, mouthpiece, high flow nasal cannula, vibrating mesh nebuliser, adult, paediatric, inhalation

## Abstract

There are variations in the values reported for aerosol drug delivery across in vitro experiments throughout the published literature, and often with the same devices or similar experimental setups. Factors contributing to this variability include, but are not limited to device type, equipment settings, drug type and quantification methods. This study assessed the impact of head model choice on aerosol drug delivery using six different adults and three different paediatric head models in combination with a facemask, mouthpiece, and high-flow nasal cannula. Under controlled test conditions, the quantity of drug collected varied depending on the choice of head model. Head models vary depending on a combination of structural design differences, facial features (size and structure), internal volume measurements and airway geometries and these variations result in the differences in aerosol delivery. Of the widely available head models used in this study, only three were seen to closely predict in vivo aerosol delivery performance in adults compared with published scintigraphy data. Further, this testing identified the limited utility of some head models under certain test conditions, for example, the range reported across head models was aerosol drug delivery of 2.62 ± 2.86% to 37.79 ± 1.55% when used with a facemask. For the first time, this study highlights the impact of head model choice on reported aerosol drug delivery within a laboratory setting and contributes to explaining the differences in values reported within the literature.

## 1. Introduction

In vitro experiments play a hugely valuable role in predicting and characterizing aerosol drug delivery across a range of device and interface combinations. The information and data reported are vital in the optimisation of device design, and a device’s clinical application. A wide range of head models is reported within the literature as a means to assess aerosol delivery to simulated spontaneously breathing adult and paediatric patients. Detailed information on make, model and material is often descriptive, but limited [1,2,3]. Reported head models for benchtop experiments vary between teaching mannequins with anatomical face and upper airways [1], airway management trainer models [4], nasal cast models [5,6], and anatomically correct face/airway models derived from CT scans [7,8,9].

It is generally accepted that 3D printed head models, generated from an individual CT scan are valuable in estimating aerosol dose delivery. Moreover, the availability of realistic airway management head models, also based on real life CT Dicom data, may further advance the sophistication of head models available in estimating aerosol drug delivery. In comparison to 3D printed head models, realistic airway management head models developed by companies, such as TruCorp (TruCorp^®^, Craigavon, Ireland) and Nasco Healthcare (Nasco Healthcare, Saugerties, NY, USA) feature anatomical landmarks, such as the teeth, tongue, nasal mucus, and skin-like surface material to closer resemble “real-life” humans. In addition, other representative head models, such as CPR and head simulation models are commonly available and utilised for aerosol drug delivery experiments. It has been our group’s experience that with the range of options available, a head model choice can be a significant contributing factor to the range of results reported across the published literature.

Despite similar experimental designs, variations exist in reports relating to aerosol drug delivery. For example, Bennett et al. [8] reported a higher respirable drug mass compared to Reminiac et al. [6] when using the same vibrating mesh nebuliser type in combination with a nasal cannula for equivalent normal and distressed breathing patterns at 30 LPM (9.63% vs. 6.70% and 15.02% vs. 10.30%), 45 LPM (6.20% vs. 3.50% and 9.88% vs. 6.70%) and 60 LPM (4.58% vs. 3.00% and 7.65% vs. 5.10%).

Contributing factors for reported variations in aerosol dose delivery include but are not limited to device type [1,6,10,11,12,13,14], supplemental gas flow rates [11,15,16,17], equipment [16], breath profiles [6,8,14], and patient interface [13,18,19,20,21,22]. The aim of this study was to assess the impact of adult and paediatric head model choice on reported aerosol drug delivery. Six adult and three paediatric head models were examined. The drug was delivered to each head model using a vibrating mesh nebuliser in combination with either a facemask, mouthpiece, or high flow nasal cannula. Additional factors with the potential to impact aerosol delivery, such as equipment type, dose quantification methodology, breath profiles, device settings and drug type were controlled throughout.

## 2. Materials and Methods

### 2.1. Head Models

**Figure 1:**
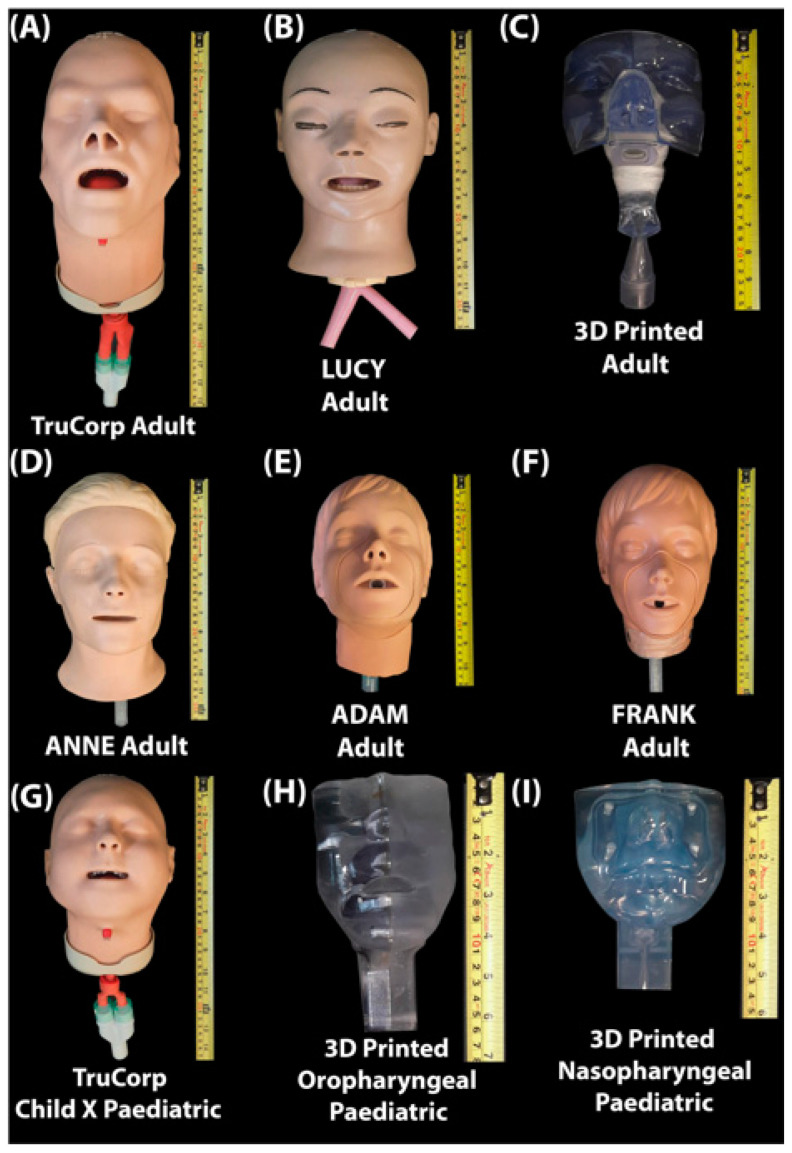
**(A**) TruCorp AirSim Advance X (AA91100X, TruCorp^®^, Craigavon, Ireland); (**B**) LUCY (Life/form^®^ Adult Airway Management Trainer, Head Only, SKU: LF03603, Nasco Healthcare, Saugerties, NY, USA); (**C**) 3D Printed anatomically correct head model generated from an individual CT scan; (**D**) ANNE (Resusci Anne^®^ QCPR, Laerdal medical, Stavanger, Norway); (**E**) FRANK (Model HSM-A 1550, Michigan Instruments, Kentwood, MI, USA); (**F**) ADAM (Adult #2000, Simulaids, INC, Saugerties, NY, USA); (**G**) TruCorp AirSim Child X (AC 10006X, TruCorp^®^, Craigavon, Ireland); (**H**) 3D Printed anatomically correct oropharyngeal (Male 5 years) generated from an individual CT scan; (**I**) 3D printed anatomically correct nasopharyngeal (1688) head model generated from an individual CT scan.

The head models included in this study are presented and described both in Figure 1 and Table 1 and consist of real-life airway management trainer models, 3D Printed head models and resuscitation and CPR models. Adult head models include two airway management trainer head models, one 3D Printed anatomically correct adult head model as previously described by Golshahi et al. [23], two CPR training head models and one head simulation model. Paediatric head models include one airway management trainer model and a 3D printed anatomically correct nasopharyngeal and oropharyngeal head model. For ease of comparison, and consistent with some reports in the literature, the head models were assigned names, see Figure 1.

External head model characteristics were assessed based on the physical characteristics and overall “real-life” representation. Factors taken into consideration include head model design and purpose, airway geometries, internal volume measurements and surface anatomical landmark dimensions, see Table 1. Measurement capabilities were subject to the structure of each head model. Nasal measurements were not recorded for the 3D printed paediatric oropharyngeal head model due to occlusion of the nasal cavity. The width of the nasal orifice was not recorded for head models ADAM or FRANK due to the lack of a nasopharyngeal airway. Oral measurements were omitted for the 3D printed adult head model due to the lack of an oral cavity.

The size, volume and structures (internal and external) varied across the different head models. The adult airway management trainer and 3D printed head models contained intricate and anatomically correct geometries. These head models offered a more “real-life” representation compared to CPR and head simulation head models (ANNE, ADAM, FRANK) which contained a single oropharyngeal airway with a simple cylindrical airway geometry.

The 3D printed paediatric nasopharyngeal and oropharyngeal head models also contained singular airways due to the lack of oral and nasal cavities. However, these airways were anatomically correct in contrast to the simplistic airways observed in the CPR and head simulation head models.

The 3D printed head models have exact replicas of nasal and airway geometries, however, they lacked additional anatomical markers, such as teeth, tongue and external facial features present on the airway management head models, LUCY (Nasco Life/form^®^ Adult Airway Management Trainer, Head Only, SKU: LF03603, Saugerties, NY, USA) and the TruCorp AirSim Advance X (AA91100X) and TruCorp AirSim Child X (AC 10006X). Similarly, the internal geometry of the airway management trainer models were generated from individual CT scans. Despite the anatomical correctness, these head models varied in shape, size and internal volume, which may be expected considering real life population heterogeneity.

The head model used depended on its compatibility with the different aerosol delivery interfaces; facemask, mouthpiece, and nasal cannula. The head models FRANK, ADAM and ANNE were not used for high flow nasal cannula testing due to the absence of a nasopharyngeal airway along with complete occlusion of the nasal cavity in FRANK and ADAM. Furthermore, ADAM was not used for mouthpiece testing due to the lack of protrusion and partial occlusion of the oral opening. The 3D printed adult head model was not structurally suitable for the attachment of a facemask and for this reason was omitted from testing using this interface. The 3D printed paediatric (Male 5 years) oropharyngeal head model was used for facemask and mouthpiece testing only due to complete occlusion of the nasal opening while the 3D printed paediatric nasopharyngeal head model was used for high flow nasal cannula testing only due to occlusion of the oral cavity. The LUCY, TruCorp AirSim Advance X and the TruCorp AirSim Child X head models were tested across all three patient interfaces considered in the study. To ensure consistency across test set-ups, the oral cavity of the TruCorp AirSim Advance Child X was occluded to align with the 3D printed nasopharyngeal (1688) paediatric head model for the nasal cannula interface experiments. Similarly, the nasal cavity of the TruCorp AirSim Advance Child X was occluded to allow for comparison with the 3D printed (Male 5 years) oropharyngeal head model for facemask and mouthpiece interface testing. Likewise, the oral cavity of the TruCorp AirSim Advance X, LUCY, and oropharyngeal airway of the 3D printed adult head model were occluded for the high flow nasal therapy testing whilst the nasal cavity of these same models was occluded during mouthpiece testing.

### 2.2. Experimental Design

**Figure 2:**
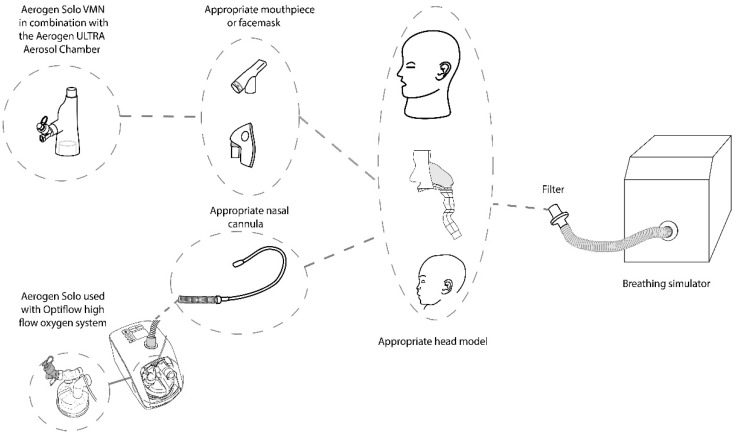
Experimental Set-up: Head model set-up in combination with the ASL 5000 breathing simulator. Aerosol administration provided with the Aerogen Ultra with a facemask or mouthpiece and the Aerogen Solo/Airvo 2 with Optiflow Nasal cannula at supplemental flow rates of 0, 2 and 6 LPM and high flow gas rates of 10 and 50 LPM respectively.

Figure 2 is an illustration of the experimental setup. Each head model was connected to a breathing simulator (ASL 5000, Ingmar medical, Pittsburgh, PA, USA) via a capture filter (303EU, Vyaire, Dublin, Ireland), placed at the level of the trachea. A breath rate (BR) of 15 breaths per minute (BPM), inspiratory to expiratory ratio (I:E) of 1:1 and tidal volume (V_t_) of 500 mL were utilised to simulate adult breath settings (ISO 27427) [24]. Appropriate settings were also applied to simulate normal paediatric breathing (V_t_: 300 mL, BR: 20 BPM, I:E 1:2).

For all tests, 4000 µg (2 mL of 2 mg/mL) Albuterol Sulfate (GlaxoSmithKline Ltd., Dublin, Ireland) was aerosolised using a vibrating mesh nebuliser (VMN) (Aerogen Solo, Aerogen, Galway, Ireland). Five test runs were completed for each head model and device interface combination.

The VMN was used in combination with an aerosol holding chamber (Aerogen Ultra, Aerogen, Ireland) and either a facemask or mouthpiece. The droplet size (VMD, Dv50) produced by the Aerogen Solo was 4.5 microns and was characterised using laser diffraction as previously described [25]. Supplemental gas flow rates of 0, 2 and 6 LPM were supplied to the Aerogen Ultra for both adult and paediatric test set ups. Although the use of the Aerogen Solo with the Ultra in a paediatric patient at supplemental gas flow rates above 2 LPM or in combination with a mouthpiece is not recommended in the manufacturer’s instructions for use in certain regions, the set-ups were used for the purposes of the current tests to investigate the impact of paediatric head model choice on aerosol delivery only.

For High Flow Nasal Therapy (HFNT) the VMN was placed at the humidifier using the Optiflow system (AIRVO 2, Fisher and Paykel Healthcare, Auckland, New Zealand). The appropriate nasal cannula (Adult: OPT946 (large), Paediatric: OPT318) and circuit (Adult: 900PT501, Paediatric: 900PT531) were used to deliver aerosol at gas flow rates of 10 & 50 LPM for simulated adult and 3, 5 & 10 LPM for simulated paediatric models, respectively.

### 2.3. Aerosol Quantification

For each test run the aerosolised drug captured on the tracheal filter (hereafter referred to as ‘tracheal dose’) was eluted from the filter using 10 mL deionised water. The drug mass was measured using UV spectroscopy at 276 nm and interpolation on a standard curve as previously described [26]. Results are expressed as a percentage (%) of the nominal dose placed in the nebuliser’s medication cup (mean ± standard deviation). Data analysis was performed using the statistical package, Minitab, version 19 (Minitab, University Park, PA, USA). A one-way ANOVA was completed for each adult test series, facemask, mouthpiece, and high flow nasal cannula. Student’s *t*-tests were completed to compare % tracheal dose between paediatric head models for facemask, mouthpiece, and high flow nasal canula testing. For all tests, differences were considered statistically significant at *p* ≤ 0.05.

## 3. Results

### 3.1. Adult Head Model Comparisons

Table 2 presents the average (±standard deviation) tracheal dose (%) (*n* = 5, for a total of 180 test runs) for each of the adult head models used in this study. The aerosol was delivered from the Aerogen Ultra to the respective head models using a facemask or mouthpiece at supplemental gas flow rates of 0, 2 and 6 LPM. Aerosol from the high flow system was delivered at 10 and 50 LPM. The data presented in Table 2 indicates that choice of head model has a statistically significant impact on aerosol drug delivery across all test settings, *p* ≤ 0.05.

### 3.2. Paediatric Head Model Comparisons

Table 3 presents the average (±standard deviation) aerosol delivered to the level of the trachea (%) (*n* = 5, for a total of 90 test runs) for the paediatric head models. Aerosol was delivered from the Aerogen Ultra at supplemental gas flow rates of 0, 2 and 6 LPM to the respective models using either a facemask or mouthpiece. Aerosol was delivered from the high flow system at flow rates 3, 5 and 10 LPM respectively via the appropriate nasal cannula. All paediatric models were constructed based on real-life CT scans, and therefore, contained anatomically correct airway geometries. The shape, size and internal volume measurements varied between head models. The 3D printed oropharyngeal head model facilitated more aerosol deposition onto the tracheal filter compared to the TruCorp AirSim Child X when used in combination with a facemask at 0 LPM (31.86 ± 3.67% vs. 5.24 ± 1.53%). In contrast, the TruCorp AirSim Child X facilitated more aerosol deposition onto the filter compared to the 3D printed oropharyngeal head model when used in combination with a mouthpiece at 0 LPM (20.42 ± 3.58% vs. 3.88 ± 0.70%). Both head models, however, were comparable when used in combination with a facemask/mouthpiece at 2 and 6 LPM. With regards to aerosol dose delivery using the high flow nasal cannula, both the TruCorp AirSim Child X and the 3D printed nasopharyngeal head model were comparable at gas flow rates of 3 and 5 LPM. However, at a flow rate of 10 LPM the 3D printed nasopharyngeal head model delivered a significantly higher percentage tracheal dose compared to the TruCorp AirSim Child X (6.28 ± 0.22% vs. 4.00 ± 0.63%). Results were considered statistically significant for *p* ≤ 0.05.

## 4. Discussion

The current findings demonstrate the impact of head model choice on aerosol drug delivery using the same device and test parameters and highlight a lack of standardisation across testing for in vitro studies in this area. Head model variation and on occasion, limited access to specific head model information impact the ability to accurately compare aerosol delivery measurements in the published literature. To our knowledge, this is the first study to assess the impact of head model choice on the assessment of aerosol drug delivery.

### 4.1. Head Model Interface Comparisons

#### 4.1.1. Facemask

Overall size and large facial features, such as the nose and mandible, influenced interface fit when using the TruCorp AirSim Advance X in combination with a facemask. It is proposed that this may have influenced the lower aerosol drug delivery measured using this head model and interface combination. Facemask fit improved and was more easily managed in smaller airway management head models (LUCY), and CPR and head simulation models (ANNE, ADAM and FRANK) (range at 0 LPM for example was 2.62 ± 2.86% to 37.79 ± 1.55%).

It is likely that the mask fit combined with both a single simplified airway geometry and small internal volume contribute to the higher percentage tracheal dose observed in FRANK when used in combination with a facemask. Aerosol is less likely to get trapped in the absence of anatomical features, such as the tongue and teeth and in the presence of simplified cylindrical airway geometries.

Although, CPR head model ADAM contained a simple airway geometry, the internal volume in this head model was larger compared to FRANK (77 cm^3^ vs. 27 cm^3^) which, along with occlusion of the nasal cavity may contribute to the lower aerosol drug delivery observed when using this model in combination with a facemask. Whilst similarly constructed, the airway geometry for ANNE contained a sharp bend as opposed to an elongated curvature of the throat as seen in FRANK and other head models. This may have influenced aerosol delivery in this head model.

In addition to interface fit it is possible that the size, structure, and presence of both the nasal and oral airways were contributing factors to aerosol dose delivery when using TruCorp AirSim Advance X and LUCY in combination with a facemask.

For paediatric head models, a small internal volume (25 cm^3^ vs. 76 cm^3^) and the presence of one (oropharyngeal) as opposed to two airways may have also enhanced aerosol delivery in the 3D printed oropharyngeal head model vs. the TruCorp AirSim Child X.

#### 4.1.2. Mouthpiece

The mouthpiece interface had a consistently good fit when used in combination with airway management trainer head models, LUCY, TruCorp AirSim Advance X, TruCorp AirSim Child X. Placing the mouthpiece directly within the oral cavity enabled direct aerosol delivery to the airways thus removing airway alignment issues present when using the facemask interface. It is possible that improved interface fit enhanced aerosol delivery for the TruCorp AirSim Advance X when used in combination with a mouthpiece compared to the facemask interface.

The oral cavity for head simulation and CPR head models FRANK and ANNE was enclosed around a simple cylindrical opening (airway). Furthermore, this opening was partially sealed and rigid, which created challenges in aligning the mouthpiece, oral opening and cylindrical airway. Similar to the facemask interface, it is likely that an altered airway geometry also impacted aerosol delivery for ANNE when used in combination with a mouthpiece.

The solid oral opening of the 3D printed oropharyngeal paediatric head model was immobile and smaller in width (3.5 cm vs. 4.0 cm) compared to the adjustable opening of the TruCorp AirSim Child X. It is possible that alignment of the mouthpiece with the airway contributed to the low delivered dose observed with this head model and interface combination when compared to the TruCorp AirSim Child X (3.88 ± 0.70% vs. 20.42 ± 3.58% at 0 LPM). This low dose increased with increasing supplemental gas flow rates, where the very low and highly variable dose at 0 LPM increased at 2 and 6 LPM and was closer to the TruCorp Air-Sim Child X. This would support the above hypothesis that aerosol entry into the oral cavity facilitated larger doses, where here, the supplemental gas flow carried the aerosol into the oral cavity, making more available for inhalation onto the tracheal filter. The structure of the 3D printed adult head model allowed direct access to the airway, which eliminated these challenges.

When considering these models’ ability to predict in vivo performance in spontaneous breathing patients, there is a paucity of direct comparisons in the literature, and only adult data is available. However, it should be noted that each of the TruCorp AirSim Advance X, LUCY and the 3D printed head model all recorded similar delivered doses to that reported in a comparative scintigraphy imaging trial in healthy adult volunteers that also used the Aerogen Ultra in combination with a mouthpiece at 0 LPM (32.40 ± 4.42%, 33.24 ± 1.64% and 30.53 ± 1.32% respectively vs. 34.1 ± 6.0%) [27].

#### 4.1.3. Nasal Cannula

With regards to high flow nasal therapy, the fit of the adult nasal cannula was visibly tighter in the LUCY and 3D printed adult head models compared to the TruCorp AirSim Advance X, which measured at least 1 cm larger in length and width of the dorsum nasi and nasal opening. The width of the nasal orifices for the two paediatric head models was similar (3D printed nasopharyngeal head model and TruCorp AirSim Child X) and thus the nasal cannula was considered a good fit in both models. Whilst the internal volume for the TruCorp AirSim Child X was larger than that for the 3D printed nasopharyngeal head model (76 cm^3^ vs. 21 cm^3^), aerosol delivery was comparable at gas flow rates of 3 and 5 LPM. The 3D printed nasopharyngeal head model delivered a significantly greater aerosol dose at a gas flow rate of 10 LPM only.

Again, the TruCorp AirSim Advance X, LUCY and the 3D printed head model all recorded comparable delivered doses (4.13 ± 1.17%, 2.60 ± 0.21% and 2.32 ± 0.23% respectively, vs. 3.46 ± 1.24%) to that reported in a scintigraphy imaging trial in healthy volunteers that used the Aerogen Solo and Optiflow system with 50 LPM gas flow rate [28].

### 4.2. Head Model Material and Design

A degree of variation is expected between head models constructed from individual CT scans, however, the anatomical correctness of these models helps predict aerosol delivery in “real-life” scenarios with greater accuracy. Combined with advanced features, such as teeth, tongue, nostril mucus and “skin”, the airway management models were more intricately representative of “real-life” patients. Nevertheless, 3D printed head models remain a reliable resource for estimating aerosol dose delivery. Javaherin et al. compared an idealised geometry with 10 realistic infant geometries and found agreement between the different models, which supports the use of an idealised 3D printed model for predicting aerosol delivery [29]. Additionally, cast models based on the plastination of human airways as previously described by Durand et al. [30] may further contribute to in vitro aerosol delivery measurements. As outlined by Le Guellec et al. [31] these cast models may have an advantage in predicting in vivo transnasal aerosol dose deposition.

Although anatomically correct, the material and construction methods for head models based on CT scans also varied. The 3D printed head model described by Golshahi et al. [23] was constructed based on an adult CT scan using a Viper SLA machine and a clear Accura 60 plastic resin material. Airway management head models, such as those available from TruCorp^®^ (TruCorp^®^, Craigavon, Ireland) are also based on CT Dicom data, however, these models are constructed of silicone skin (externally) and latex based airways (internally). It is possible that the material composition and construction methods may further influence aerosol delivery through the airways of varying head models.

Not addressed by these models, and consequently by our analysis, are several features that exist in humans, such as the variable position of the tongue, jaw and glottal aperture. This, combined with the effect of the airway lining liquids and mucus will impact aerosol deposition, and ultimately aerosol distribution throughout the airways. Additionally, these upper airway models, and despite some being derived from CT scans, are not representative of the entire human adult or paediatric populations. As such, reported aerosol doses should always be considered indicative and no more. Attempts have been made to address these limitations in models as tools, but there are still none that meet all the needs of the research field. For example, the Alberta Idealized Throat model [32] was developed as a composite from several CT scans, however, it lacks the nasopharynx and face. In paediatric modelling, the SAINT model of a 9-month-old [33] was derived from a single CT scan and made use of a bespoke airway coating solution but whilst it has facial features, again it is nasopharyngeal only. The oropharynx is not included.

Furthermore, as noted above, interface (mask/mouthpiece/cannula) fit against the face/head model is a critical determinant of aerosol delivery. Whilst not the focus of our studies, this interface “fit” element is deserving of further investigation in future studies. This may lead to complexity in interface options, e.g., several sizes need to be designed or made available, but the resulting improvement in aerosol delivery and the consequent healthcare outcomes are deserving reasons.

A considerable amount of work is required in order to close these gaps but until that happens, the commercially available head models, such as those described herein remain a valuable research tool, albeit with their own limitations.

## 5. Conclusions

A range of commercially available head models is described within studies pertaining to aerosol inhalation and drug delivery across the published literature. These head models vary depending on design, structure, volume, and geometrical complexities and, thus, impact aerosol measurements when used in combination with a range of interfaces. With significant variability noted across combinations of head model type, interface and supplemental gas flow rate, this study highlights the need for increased awareness on the impact of head model choice on reported aerosol drug delivery. As such, the suitability of and ability to predict in vivo aerosol performance of the head model under test needs to be considered carefully. Here, based on limited in vivo data reported in the literature, we found the TruCorp AirSim Advance X, LUCY and the 3D printed head model the most predictive of in vivo aerosol delivery performance for both mouthpiece and nasal cannula in a model of the adult patient. Unfortunately, due to the absence of published scintigraphy data using the same nebuliser in paediatric patients, we were unable to determine the most predictive of in vivo performance in a model of the paediatric patient. Comparisons with reported nonhuman primate data where the same nebuliser was used, for example, Réminiac et al. [20] are not appropriate given the significant anatomical differences. This remains a gap in the state of the art, especially when one considers the variability in dose across the three paediatric head models reported herein. Finally, similar to breathing pattern, nebuliser type and patient intervention, we recommend that head model type should be documented clearly and carefully across in vitro benchtop studies in order to ensure that findings can be both reproduced and interpreted correctly in cases where a model may have been unsuitable for use under certain test conditions.

## Figures and Tables

**Table 1:** Head Model Characteristics.

	Head Model	Design	Internal Geometry	Internal Volume	Dorsum Nasi (L) ^1^	Nasal Orifices (w) ^1^	Oral Opening (w) ^1^
Adult	LUCY	Airway management trainer model	Nasal and oral anatomical landmarks	212 cm^3^	3.5 cm	2.2 cm	4.5 cm
	TruCorp AirSim Advance X	Airway management trainer model	Nasal and oral anatomical landmarks	318 cm^3^	4.5 cm	3.5 cm	5.0 cm
	ANNE	CPR Trainer Model	Simple single airway	52 cm^3^	4.3 cm	2.0 cm	5.0 cm
	ADAM	CPR Adult Manikin	Simple single airway	77 cm^3^	4.3 cm	N/A	4.5 cm
	FRANK	Head Simulation Model	Simple single airway	27 cm^3^	4.3 cm	N/A	4.5 cm
	3D printed Adult Head Model	3D printed generated from individual CT scan	Anatomically correct geometry	84 cm^3^	3.5 cm	2.5 cm	N/A
Paediatric	TruCorp AirSim Child X	Airway management trainer model	Anatomically correct geometry	76 cm^3^	3.4 cm	2.2 cm	4.0 cm
	3D printed Paediatric oropharyngeal head model	3D printed generated from individual CT scan	Single anatomically correct oropharyngeal airway	25 cm^3^	N/A	N/A	3.5 cm
	3D printed nasopharyngeal head model	3D printed Generated from individual CT scan	Single anatomically correct nasopharyngeal airway	21 cm^3^	2.0 cm	2.3 cm	N/A

^1^ Nasal surface measurements represent the length of the Dorsum Nasi (L) and width of the lateral openings (W) of the nasal and oral orifices.

**Table 2:** Mean ± SD % tracheal dose for Adult head models in combination with the Aerogen Ultra with a facemask or mouthpiece and the Aerogen Solo/Airvo 2 with Optiflow Nasal cannula at supplemental flow rates of 0, 2 and 6 LPM and high flow gas rates of 10 and 50 LPM respectively.

Interface	Supplemental Gas Flow Rate	TruCorp AirSim Advance X	LUCY	ANNE	ADAM	FRANK	3D Printed Adult Head Model	*p*-Value
Facemask	0	6.35 ± 0.87	22.79 ± 1.84	2.62 ± 2.86	13.02 ± 1.60	37.79 ± 1.55	NT ^1^	0.000
	2	17.48 ± 1.82	29.30 ± 0.95	24.89 ± 3.65	16.61 ± 2.04	35.80 ± 1.26	NT ^1^	0.000
	6	16.75 ± 2.86	23.61 ± 1.73	24.18 ± 3.07	17.55 ± 1.82	31.25 ± 2.54	NT ^1^	0.000
Mouthpiece	0	32.40 ± 4.42	33.24 ± 1.64	1.93 ± 2.79	NT ^1^	28.20 ± 1.33	30.53 ± 1.32	0.000
	2	36.56 ± 4.13	32.90 ± 2.95	27.94 ± 2.49	NT ^1^	30.10 ± 1.37	30.54 ± 3.37	0.003
	6	22.29 ± 1.42	21.92 ± 1.92	28.11 ± 2.13	NT ^1^	24.97 ± 1.19	24.96 ± 3.74	0.002
Nasal Cannula	10	11.85 ± 1.07	15.97 ± 1.84	NT ^1^	NT ^1^	NT ^1^	15.66 ± 0.87	0.001
	50	4.13 ± 1.17	2.60 ± 0.21	NT ^1^	NT ^1^	NT ^1^	2.32 ± 0.23	0.003

^1^ NT = not tested.

**Table 3:** Mean ± SD % tracheal dose for Paediatric Head Models in combination with the Aerogen Ultra with a facemask or mouthpiece and the Aerogen Solo/Airvo 2 with Optiflow Nasal cannula at supplemental flow rates of 0, 2 and 6 LPM and high flow gas rates of 3, 5 and 10 LPM respectively.

Interface	Supplemental Gas Flow Rate (LPM)	TruCorp AirSim Child X	3D Printed Oropharyngeal Head Model	3D Printed Nasopharyngeal Head Model	*p*-Value
Facemask	0	5.24 ± 1.53	31.86 ± 3.67	NT ^1^	0.000
	2	21.51 ± 4.14	24.05 ± 5.88	NT ^1^	0.453
	6	25.50 ± 4.94	26.94 ± 2.73	NT ^1^	0.583
Mouthpiece	0	20.42 ± 3.58	3.88 ± 0.70	NT ^1^	0.000
	2	23.43 ± 3.99	26.48 ± 7.76	NT ^1^	0.457
	6	21.18 ± 0.56	24.03 ± 3.62	NT ^1^	0.120
Nasal Cannula	3	9.08 ± 2.67	NT ^1^	6.49 ± 2.46	0.149
	5	7.04 ± 1.46	NT ^1^	5.55 ± 0.98	0.094
	10	4.00 ± 0.63	NT ^1^	6.28 ± 0.22	0.000

^1^ NT = not tested.

## Data Availability

The data presented in this study are available on request from the corresponding author.
